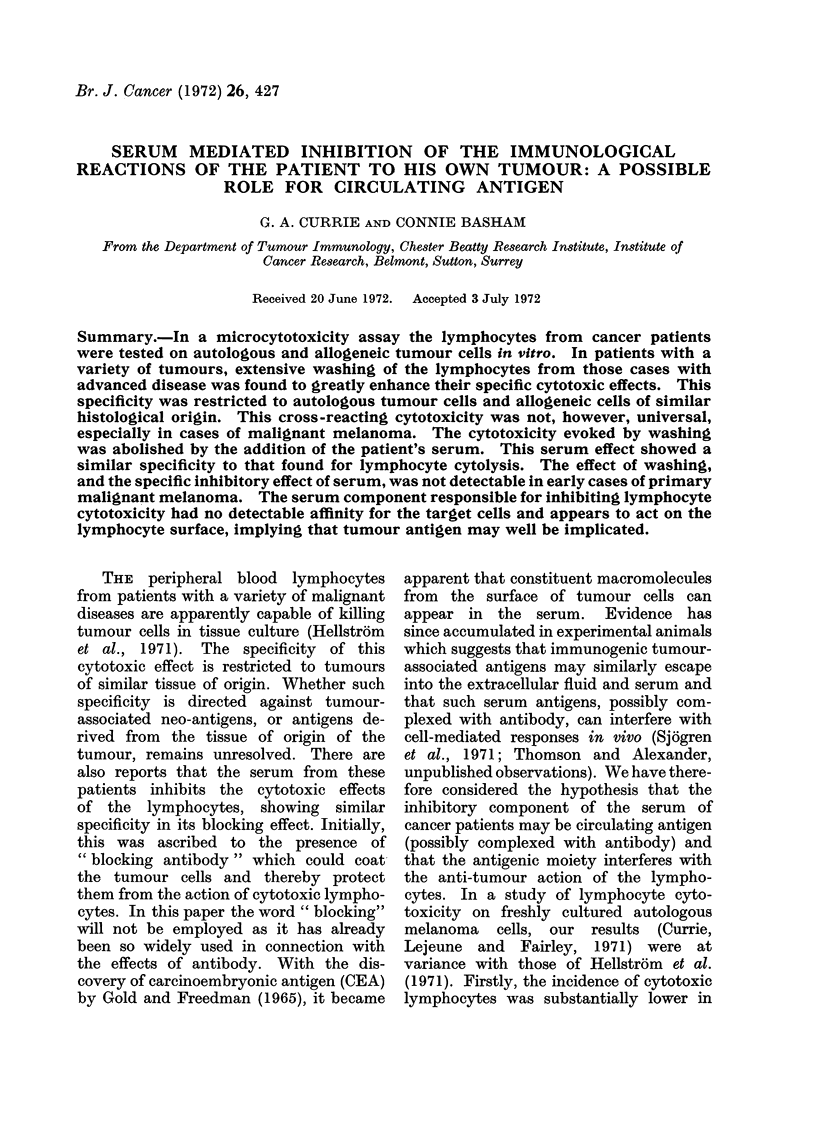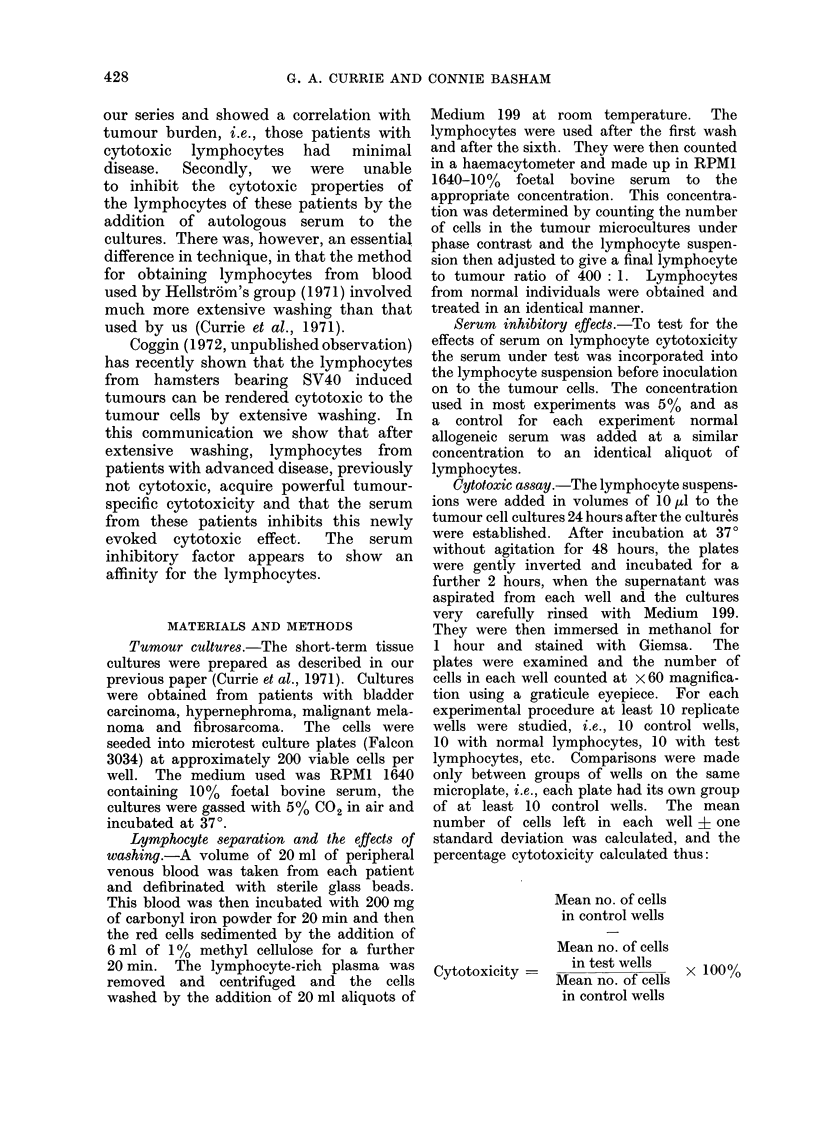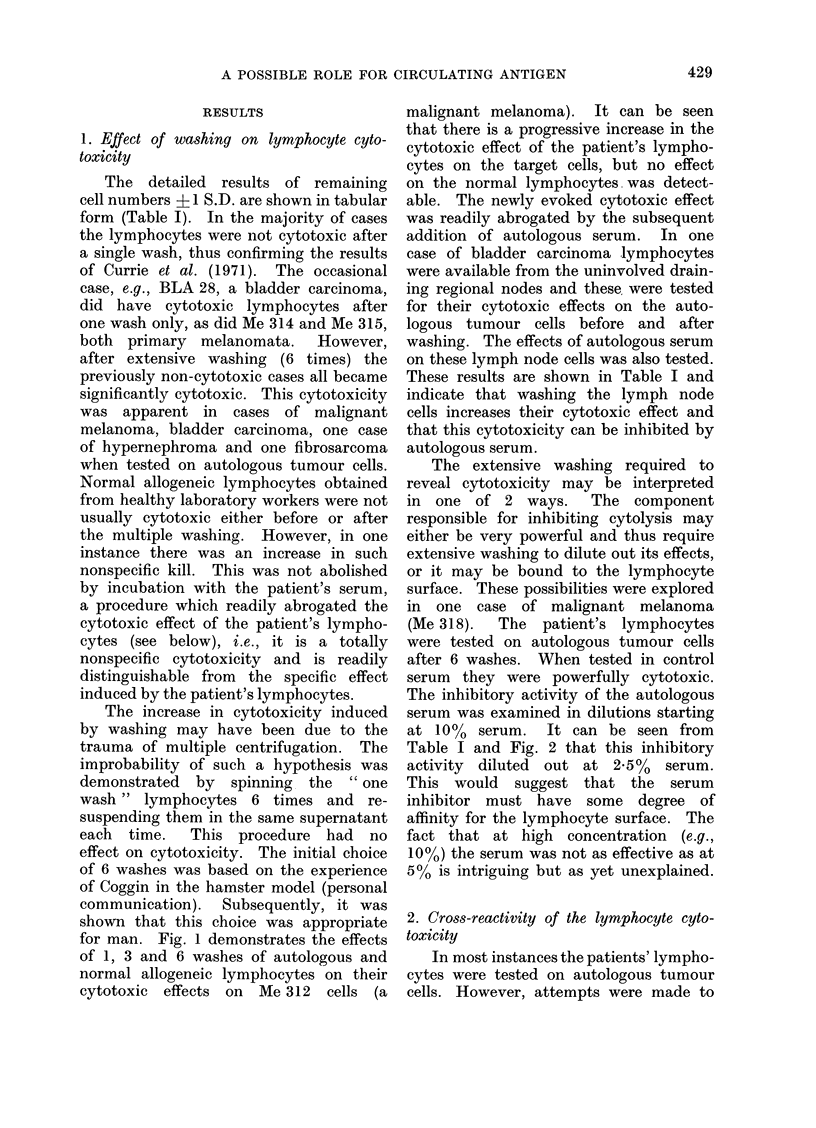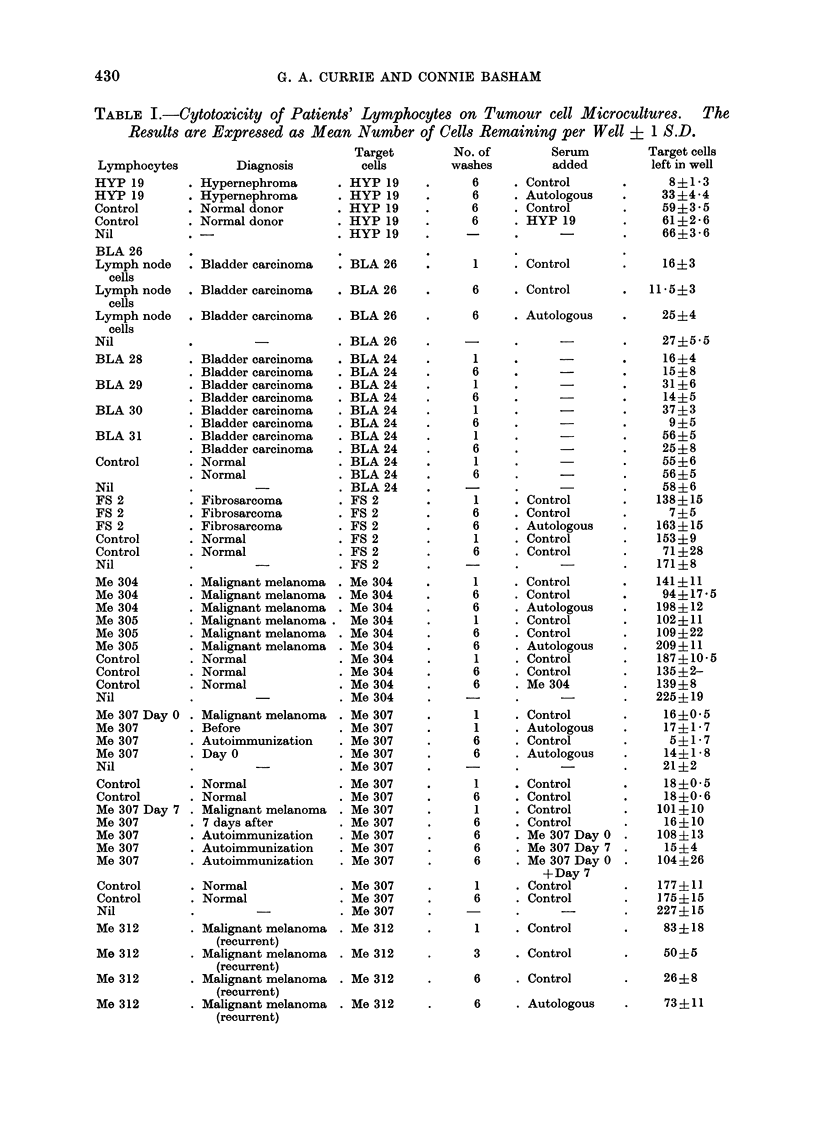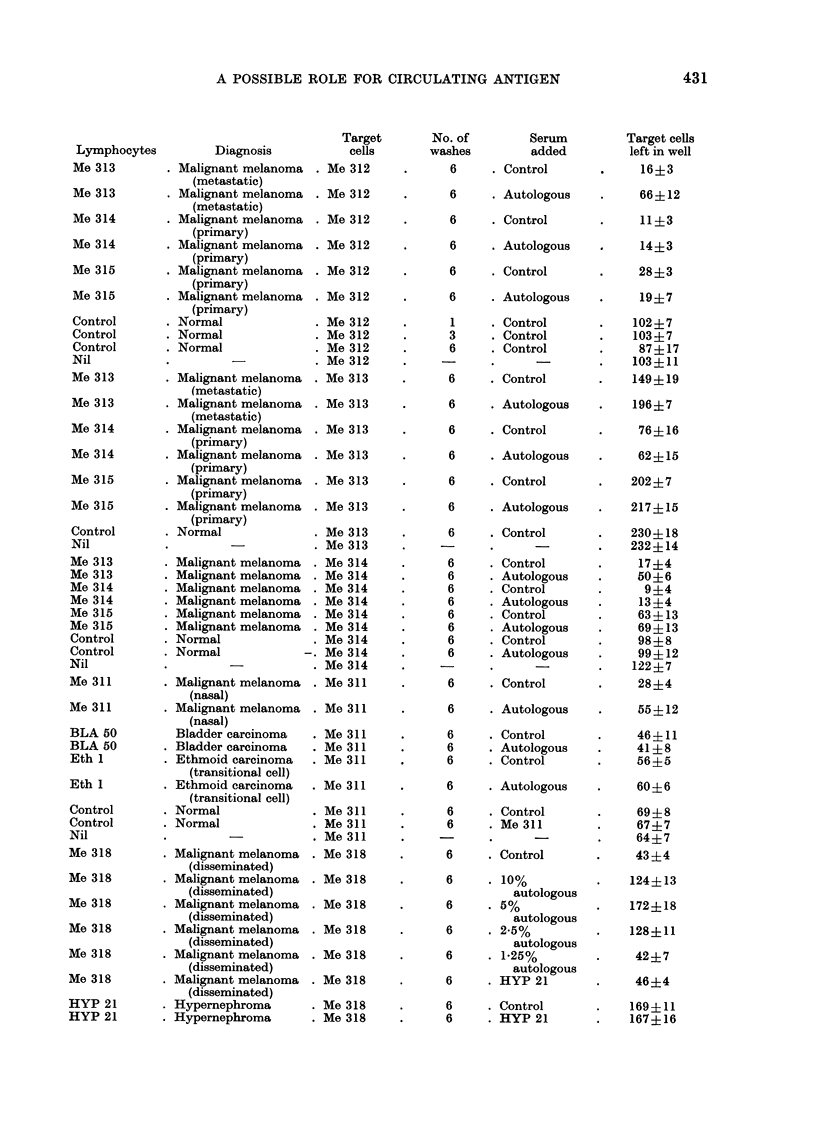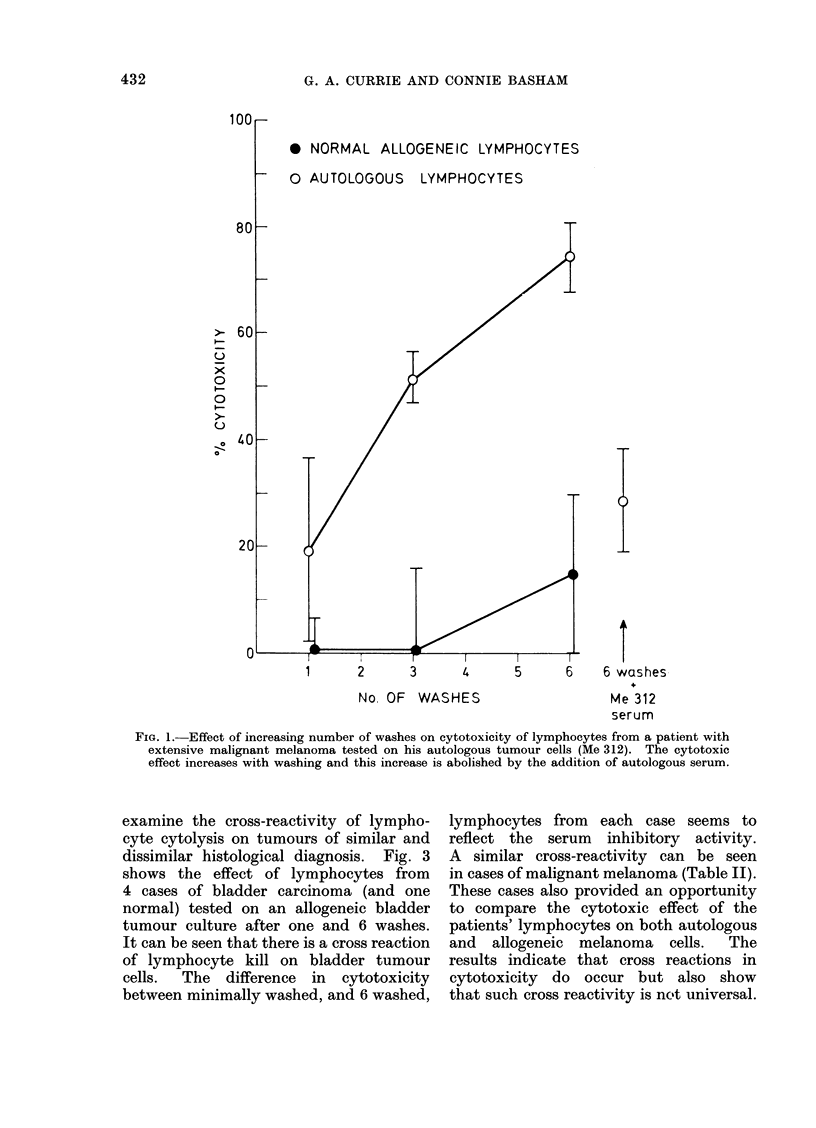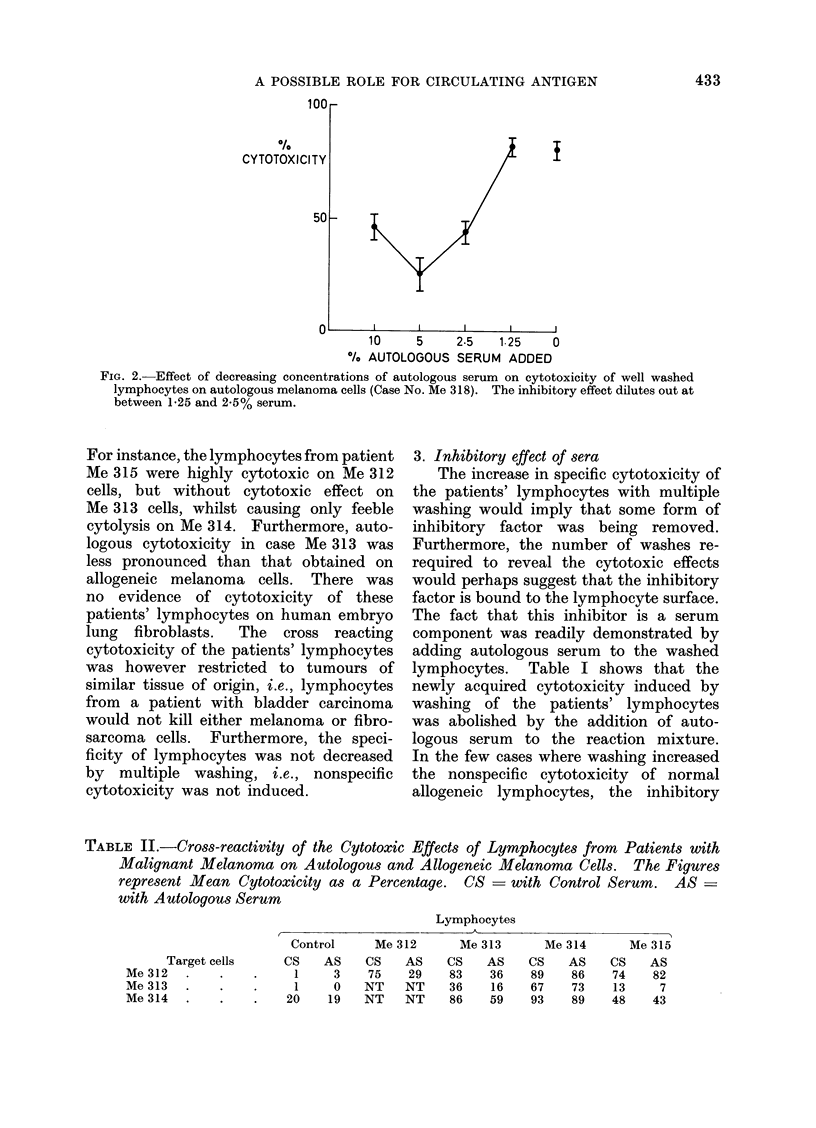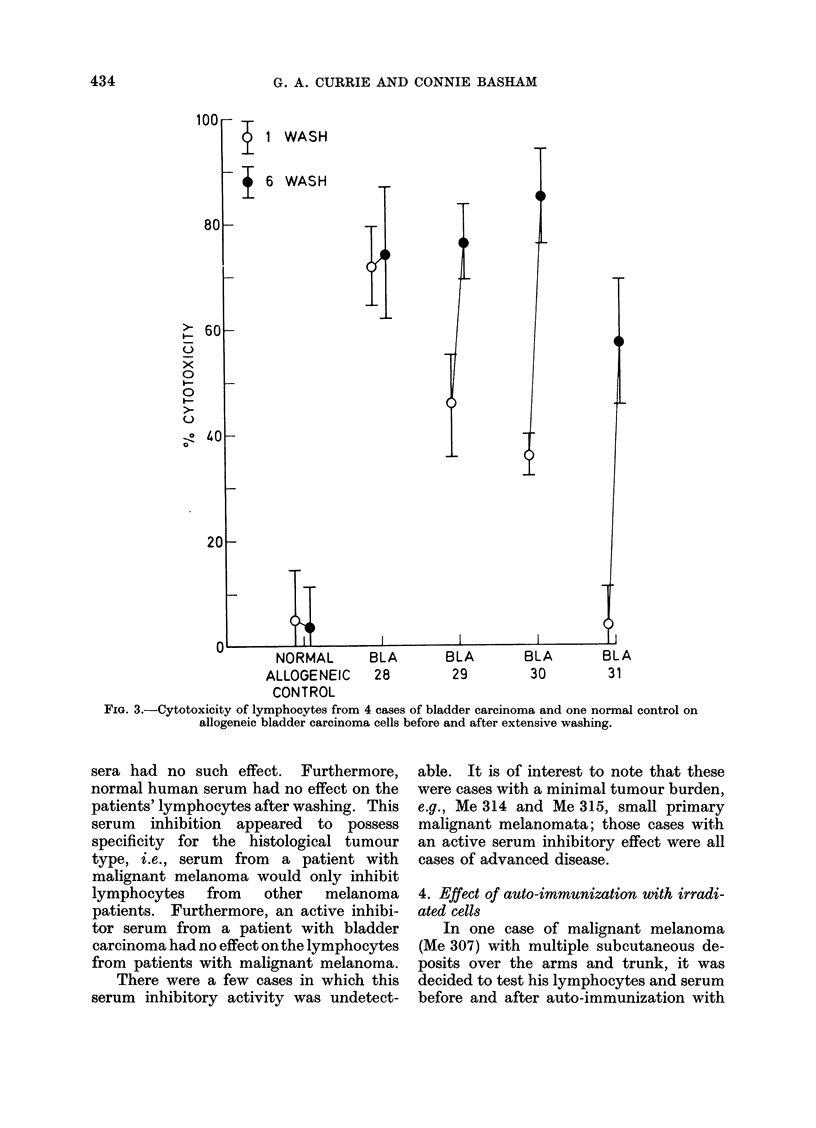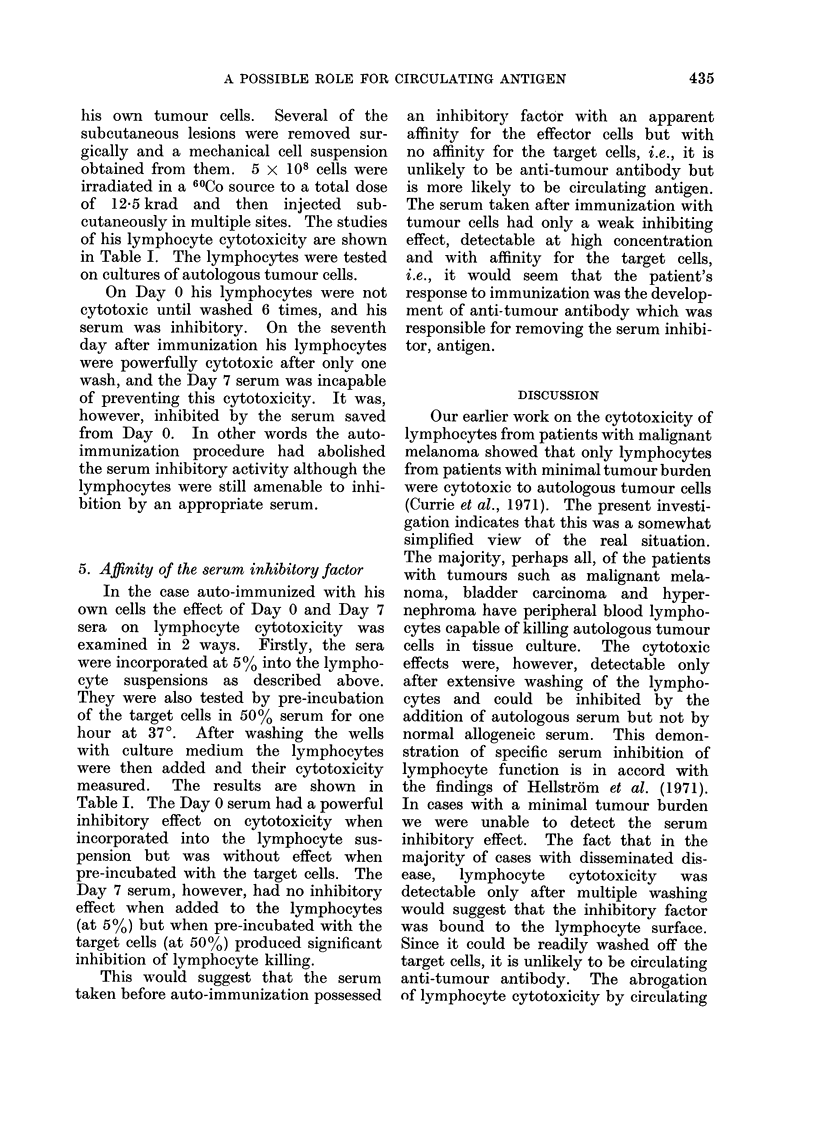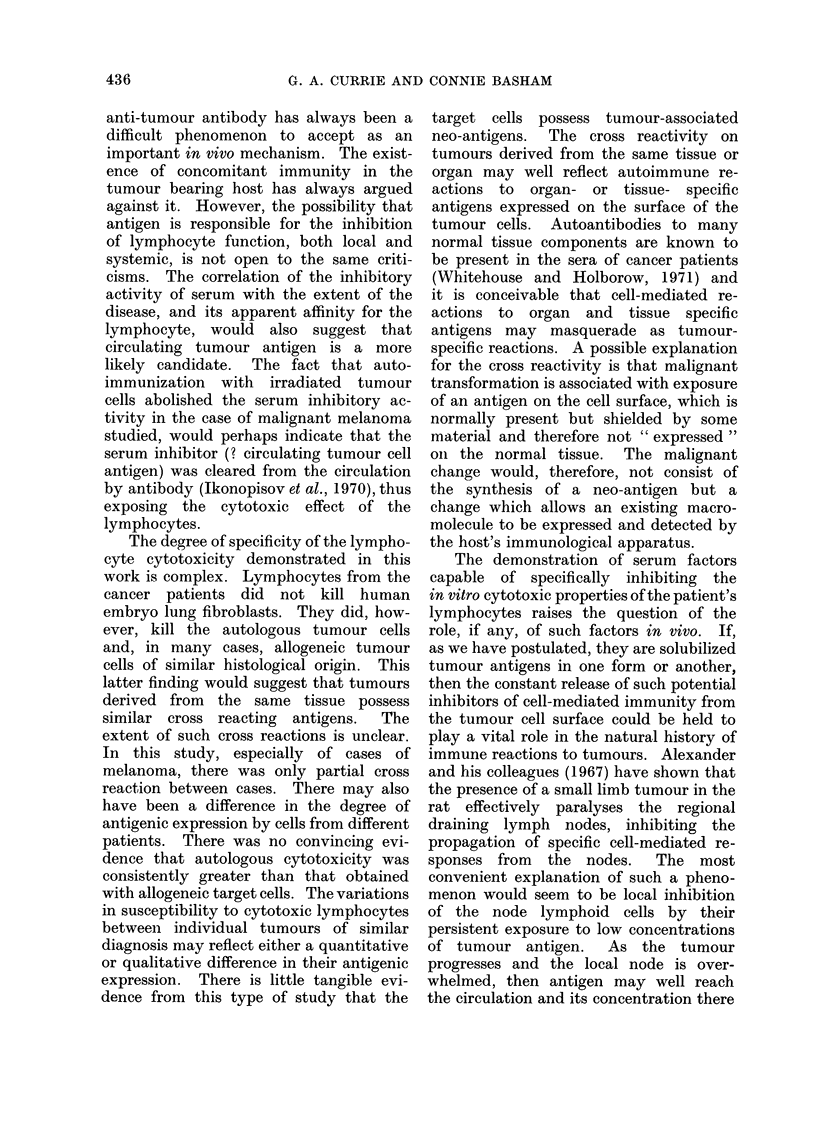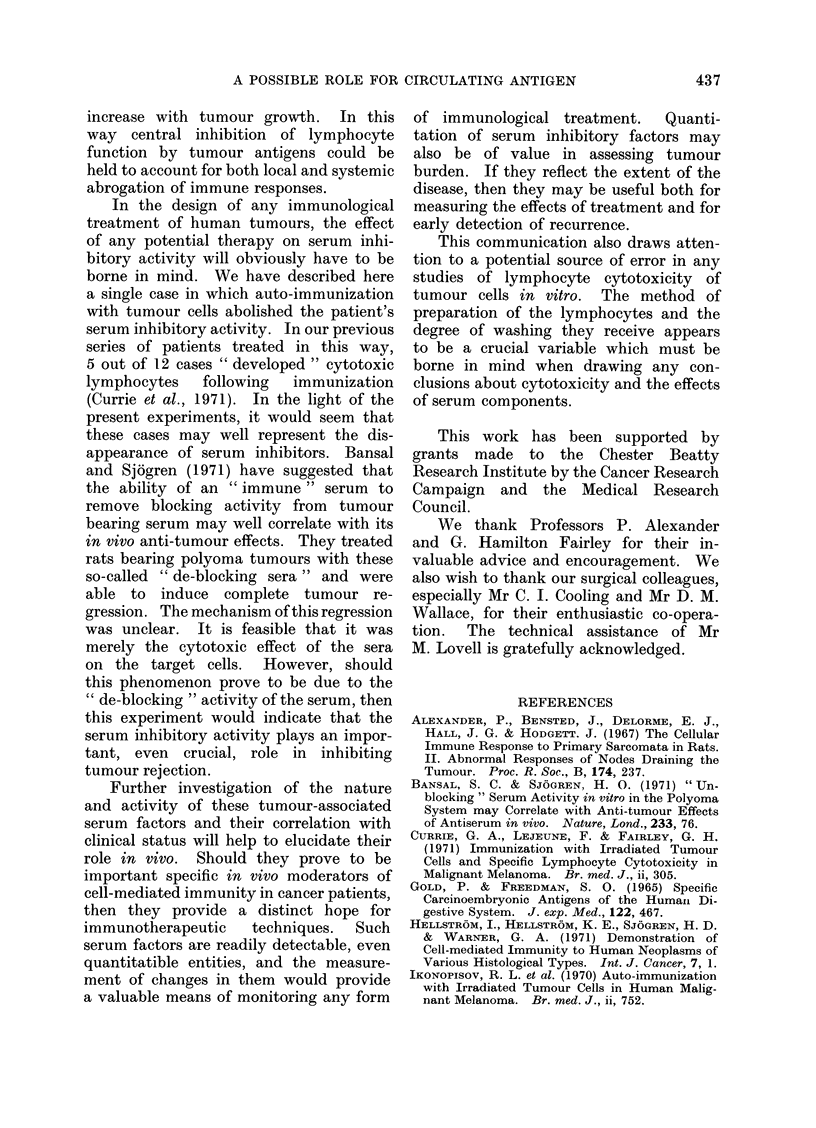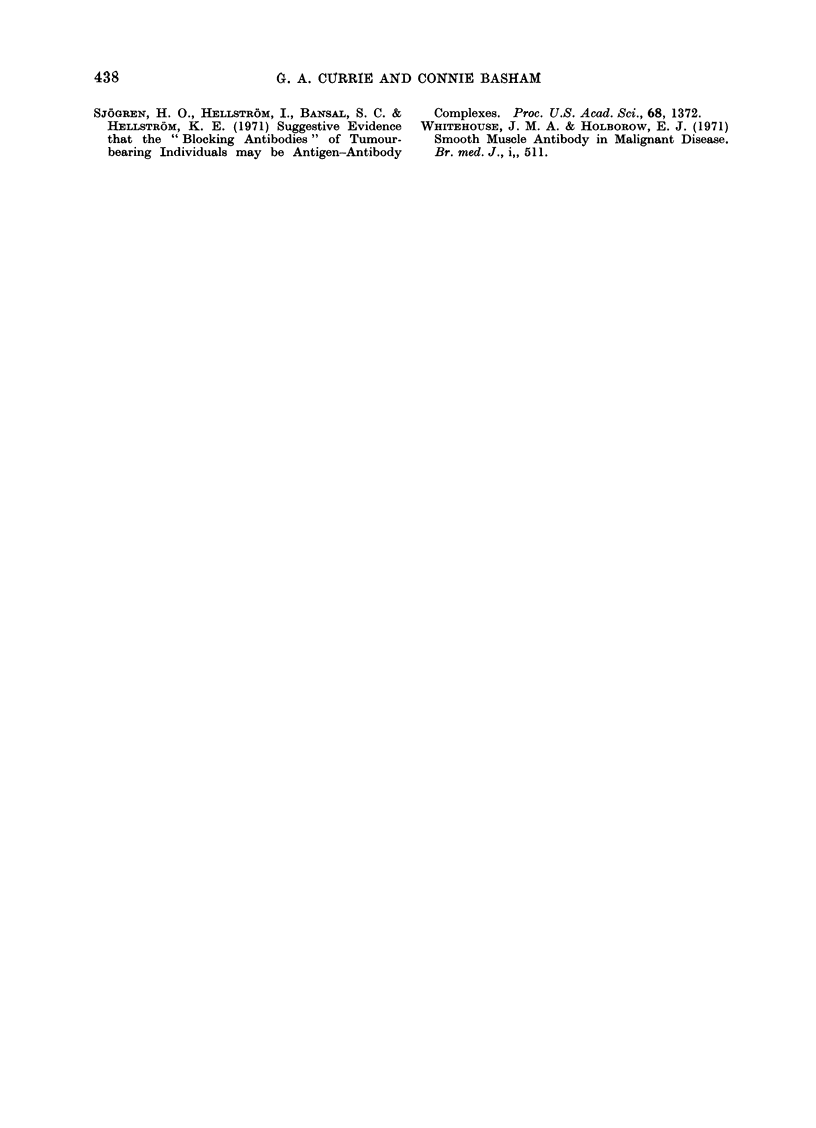# Serum Mediated Inhibition of the Immunological Reactions of the Patient to his Own Tumour: A Possible Role for Circulating Antigen

**DOI:** 10.1038/bjc.1972.59

**Published:** 1972-12

**Authors:** G. A. Currie, Connie Basham

## Abstract

In a microcytotoxicity assay the lymphocytes from cancer patients were tested on autologous and allogeneic tumour cells *in vitro.* In patients with a variety of tumours, extensive washing of the lymphocytes from those cases with advanced disease was found to greatly enhance their specific cytotoxic effects. This specificity was restricted to autologous tumour cells and allogeneic cells of similar histological origin. This cross-reacting cytotoxicity was not, however, universal, especially in cases of malignant melanoma. The cytotoxicity evoked by washing was abolished by the addition of the patient's serum. This serum effect showed a similar specificity to that found for lymphocyte cytolysis. The effect of washing, and the specific inhibitory effect of serum, was not detectable in early cases of primary malignant melanoma. The serum component responsible for inhibiting lymphocyte cytotoxicity had no detectable affinity for the target cells and appears to act on the lymphocyte surface, implying that tumour antigen may well be implicated.


*Br. J. Cancer* (1972) **26**, 427

### SERUM MEDIATED INHIBITION OF THE IMMUNOLOGICAL REACTIONS OF THE PATIENT TO HIS OWN TUMOUR: A POSSIBLE ROLE FOR CIRCULATING ANTIGEN

G. A. CURRIE AND CONNIE BASHAM

*From the Department of Tumour Immunology, Chester Beatty Research Institute, Institute of Cancer Research, Belmont, Sutton, Surrey*

Received 20 June 1972. Accepted 3 July 1972

**Summary.—In a microcytotoxicity assay the lymphocytes from cancer patients were tested on autologous and allogeneic tumour cells *in vitro*. In patients with a variety of tumours, extensive washing of the lymphocytes from those cases with advanced disease was found to greatly enhance their specific cytotoxic effects. This specificity was restricted to autologous tumour cells and allogeneic cells of similar histological origin. This cross-reacting cytotoxicity was not, however, universal, especially in cases of malignant melanoma. The cytotoxicity evoked by washing was abolished by the addition of the patient's serum. This serum effect showed a similar specificity to that found for lymphocyte cytolysis. The effect of washing, and the specific inhibitory effect of serum, was not detectable in early cases of primary malignant melanoma. The serum component responsible for inhibiting lymphocyte cytotoxicity had no detectable affinity for the target cells and appears to act on the lymphocyte surface, implying that tumour antigen may well be implicated.**

THE peripheral blood lymphocytes from patients with a variety of malignant diseases are apparently capable of killing tumour cells in tissue culture (Hellström *et al.*, 1971). The specificity of this cytotoxic effect is restricted to tumours of similar tissue of origin. Whether such specificity is directed against tumour-associated neo-antigens, or antigens derived from the tissue of origin of the tumour, remains unresolved. There are also reports that the serum from these patients inhibits the cytotoxic effects of the lymphocytes, showing similar specificity in its blocking effect. Initially, this was ascribed to the presence of " blocking antibody " which could coat the tumour cells and thereby protect them from the action of cytotoxic lymphocytes. In this paper the word " blocking" will not be employed as it has already been so widely used in connection with the effects of antibody. With the discovery of carcinoembryonic antigen (CEA) by Gold and Freedman (1965), it became apparent that constituent macromolecules from the surface of tumour cells can appear in the serum. Evidence has since accumulated in experimental animals which suggests that immunogenic tumour-associated antigens may similarly escape into the extracellular fluid and serum and that such serum antigens, possibly complexed with antibody, can interfere with cell-mediated responses *in vivo* (Sjögren *et al.*, 1971; Thomson and Alexander, unpublished observations). We have therefore considered the hypothesis that the inhibitory component of the serum of cancer patients may be circulating antigen (possibly complexed with antibody) and that the antigenic moiety interferes with the anti-tumour action of the lymphocytes. In a study of lymphocyte cytotoxicity on freshly cultured autologous melanoma cells, our results (Currie, Lejeune and Fairley, 1971) were at variance with those of Hellström *et al.* (1971). Firstly, the incidence of cytotoxic lymphocytes was substantially lower in our series and showed a correlation with tumour burden, *i.e.*, those patients with cytotoxic lymphocytes had minimal disease. Secondly, we were unable to inhibit the cytotoxic properties of the lymphocytes of these patients by the addition of autologous serum to the cultures. There was, however, an essential difference in technique, in that the method for obtaining lymphocytes from blood used by Hellström's group (1971) involved much more extensive washing than that used by us (Currie *et al.*, 1971).

Coggin (1972, unpublished observation) has recently shown that the lymphocytes from hamsters bearing SV40 induced tumours can be rendered cytotoxic to the tumour cells by extensive washing. In this communication we show that after extensive washing, lymphocytes from patients with advanced disease, previously not cytotoxic, acquire powerful tumour-specific cytotoxicity and that the serum from these patients inhibits this newly evoked cytotoxic effect. The serum inhibitory factor appears to show an affinity for the lymphocytes.

### MATERIALS AND METHODS

*Tumour cultures.*—The short-term tissue cultures were prepared as described in our previous paper (Currie *et al.*, 1971). Cultures were obtained from patients with bladder carcinoma, hypernephroma, malignant melanoma and fibrosarcoma. The cells were seeded into microtest culture plates (Falcon 3034) at approximately 200 viable cells per well. The medium used was RPM1 1640 containing 10% foetal bovine serum, the cultures were gassed with 5% $CO_2$ in air and incubated at 37°.

*Lymphocyte separation and the effects of washing.*—A volume of 20 ml of peripheral venous blood was taken from each patient and defibrinated with sterile glass beads. This blood was then incubated with 200 mg of carbonyl iron powder for 20 min and then the red cells sedimented by the addition of 6 ml of 1% methyl cellulose for a further 20 min. The lymphocyte-rich plasma was removed and centrifuged and the cells washed by the addition of 20 ml aliquots of Medium 199 at room temperature. The lymphocytes were used after the first wash and after the sixth. They were then counted in a haemacytometer and made up in RPM1 1640–10% foetal bovine serum to the appropriate concentration. This concentration was determined by counting the number of cells in the tumour microcultures under phase contrast and the lymphocyte suspension then adjusted to give a final lymphocyte to tumour ratio of 400 : 1. Lymphocytes from normal individuals were obtained and treated in an identical manner.

*Serum inhibitory effects.*—To test for the effects of serum on lymphocyte cytotoxicity the serum under test was incorporated into the lymphocyte suspension before inoculation on to the tumour cells. The concentration used in most experiments was 5% and as a control for each experiment normal allogeneic serum was added at a similar concentration to an identical aliquot of lymphocytes.

*Cytotoxic assay.*—The lymphocyte suspensions were added in volumes of 10 $\mu$l to the tumour cell cultures 24 hours after the cultures were established. After incubation at 37° without agitation for 48 hours, the plates were gently inverted and incubated for a further 2 hours, when the supernatant was aspirated from each well and the cultures very carefully rinsed with Medium 199. They were then immersed in methanol for 1 hour and stained with Giemsa. The plates were examined and the number of cells in each well counted at ×60 magnification using a graticule eyepiece. For each experimental procedure at least 10 replicate wells were studied, *i.e.*, 10 control wells, 10 with normal lymphocytes, 10 with test lymphocytes, etc. Comparisons were made only between groups of wells on the same microplate, *i.e.*, each plate had its own group of at least 10 control wells. The mean number of cells left in each well ± one standard deviation was calculated, and the percentage cytotoxicity calculated thus:

$$\text{Cytotoxicity} = \frac{\begin{matrix}\text{Mean no. of cells}\\ \text{in control wells}\\ -\\ \text{Mean no. of cells}\\ \text{in test wells}\end{matrix}}{\begin{matrix}\text{Mean no. of cells}\\ \text{in control wells}\end{matrix}} \times 100\%$$

### RESULTS

### 1. *Effect of washing on lymphocyte cytotoxicity*

The detailed results of remaining cell numbers $\pm 1$ S.D. are shown in tabular form (Table I). In the majority of cases the lymphocytes were not cytotoxic after a single wash, thus confirming the results of Currie *et al.* (1971). The occasional case, *e.g.*, BLA 28, a bladder carcinoma, did have cytotoxic lymphocytes after one wash only, as did Me 314 and Me 315, both primary melanomata. However, after extensive washing (6 times) the previously non-cytotoxic cases all became significantly cytotoxic. This cytotoxicity was apparent in cases of malignant melanoma, bladder carcinoma, one case of hypernephroma and one fibrosarcoma when tested on autologous tumour cells. Normal allogeneic lymphocytes obtained from healthy laboratory workers were not usually cytotoxic either before or after the multiple washing. However, in one instance there was an increase in such nonspecific kill. This was not abolished by incubation with the patient's serum, a procedure which readily abrogated the cytotoxic effect of the patient's lymphocytes (see below), *i.e.*, it is a totally nonspecific cytotoxicity and is readily distinguishable from the specific effect induced by the patient's lymphocytes.

The increase in cytotoxicity induced by washing may have been due to the trauma of multiple centrifugation. The improbability of such a hypothesis was demonstrated by spinning the "one wash" lymphocytes 6 times and resuspending them in the same supernatant each time. This procedure had no effect on cytotoxicity. The initial choice of 6 washes was based on the experience of Coggin in the hamster model (personal communication). Subsequently, it was shown that this choice was appropriate for man. Fig. 1 demonstrates the effects of 1, 3 and 6 washes of autologous and normal allogeneic lymphocytes on their cytotoxic effects on Me 312 cells (a malignant melanoma). It can be seen that there is a progressive increase in the cytotoxic effect of the patient's lymphocytes on the target cells, but no effect on the normal lymphocytes was detectable. The newly evoked cytotoxic effect was readily abrogated by the subsequent addition of autologous serum. In one case of bladder carcinoma lymphocytes were available from the uninvolved draining regional nodes and these were tested for their cytotoxic effects on the autologous tumour cells before and after washing. The effects of autologous serum on these lymph node cells was also tested. These results are shown in Table I and indicate that washing the lymph node cells increases their cytotoxic effect and that this cytotoxicity can be inhibited by autologous serum.

The extensive washing required to reveal cytotoxicity may be interpreted in one of 2 ways. The component responsible for inhibiting cytolysis may either be very powerful and thus require extensive washing to dilute out its effects, or it may be bound to the lymphocyte surface. These possibilities were explored in one case of malignant melanoma (Me 318). The patient's lymphocytes were tested on autologous tumour cells after 6 washes. When tested in control serum they were powerfully cytotoxic. The inhibitory activity of the autologous serum was examined in dilutions starting at 10% serum. It can be seen from Table I and Fig. 2 that this inhibitory activity diluted out at 2·5% serum. This would suggest that the serum inhibitor must have some degree of affinity for the lymphocyte surface. The fact that at high concentration (*e.g.*, 10%) the serum was not as effective as at 5% is intriguing but as yet unexplained.

### 2. *Cross-reactivity of the lymphocyte cytotoxicity*

In most instances the patients' lymphocytes were tested on autologous tumour cells. However, attempts were made to

TABLE I.—*Cytotoxicity of Patients' Lymphocytes on Tumour cell Microcultures. The Results are Expressed as Mean Number of Cells Remaining per Well ± 1 S.D.*

| Lymphocytes | Diagnosis | Target cells | No. of washes | Serum added | Target cells left in well |
|---|---|---|---|---|---|
| HYP 19 | Hypernephroma | HYP 19 | 6 | Control | 8±1·3 |
| HYP 19 | Hypernephroma | HYP 19 | 6 | Autologous | 33±4·4 |
| Control | Normal donor | HYP 19 | 6 | Control | 59±3·5 |
| Control | Normal donor | HYP 19 | 6 | HYP 19 | 61±2·6 |
| Nil | — | HYP 19 | — | — | 66±3·6 |
| BLA 26 | | | | | |
| Lymph node cells | Bladder carcinoma | BLA 26 | 1 | Control | 16±3 |
| Lymph node cells | Bladder carcinoma | BLA 26 | 6 | Control | 11·5±3 |
| Lymph node cells | Bladder carcinoma | BLA 26 | 6 | Autologous | 25±4 |
| Nil | — | BLA 26 | — | — | 27±5·5 |
| BLA 28 | Bladder carcinoma | BLA 24 | 1 | — | 16±4 |
| | Bladder carcinoma | BLA 24 | 6 | — | 15±8 |
| BLA 29 | Bladder carcinoma | BLA 24 | 1 | — | 31±6 |
| | Bladder carcinoma | BLA 24 | 6 | — | 14±5 |
| BLA 30 | Bladder carcinoma | BLA 24 | 1 | — | 37±3 |
| | Bladder carcinoma | BLA 24 | 6 | — | 9±5 |
| BLA 31 | Bladder carcinoma | BLA 24 | 1 | — | 56±5 |
| | Bladder carcinoma | BLA 24 | 6 | — | 25±8 |
| Control | Normal | BLA 24 | 1 | — | 55±6 |
| | Normal | BLA 24 | 6 | — | 56±5 |
| Nil | — | BLA 24 | — | — | 58±6 |
| FS 2 | Fibrosarcoma | FS 2 | 1 | Control | 138±15 |
| FS 2 | Fibrosarcoma | FS 2 | 6 | Control | 7±5 |
| FS 2 | Fibrosarcoma | FS 2 | 6 | Autologous | 163±15 |
| Control | Normal | FS 2 | 1 | Control | 153±9 |
| Control | Normal | FS 2 | 6 | Control | 71±28 |
| Nil | — | FS 2 | — | — | 171±8 |
| Me 304 | Malignant melanoma | Me 304 | 1 | Control | 141±11 |
| Me 304 | Malignant melanoma | Me 304 | 6 | Control | 94±17·5 |
| Me 304 | Malignant melanoma | Me 304 | 6 | Autologous | 198±12 |
| Me 305 | Malignant melanoma | Me 304 | 1 | Control | 102±11 |
| Me 305 | Malignant melanoma | Me 304 | 6 | Control | 109±22 |
| Me 305 | Malignant melanoma | Me 304 | 6 | Autologous | 209±11 |
| Control | Normal | Me 304 | 1 | Control | 187±10·5 |
| Control | Normal | Me 304 | 6 | Control | 135±2– |
| Control | Normal | Me 304 | 6 | Me 304 | 139±8 |
| Nil | — | Me 304 | — | — | 225±19 |
| Me 307 Day 0 | Malignant melanoma | Me 307 | 1 | Control | 16±0·5 |
| Me 307 | Before | Me 307 | 1 | Autologous | 17±1·7 |
| Me 307 | Autoimmunization | Me 307 | 6 | Control | 5±1·7 |
| Me 307 | Day 0 | Me 307 | 6 | Autologous | 14±1·8 |
| Nil | — | Me 307 | — | — | 21±2 |
| Control | Normal | Me 307 | 1 | Control | 18±0·5 |
| Control | Normal | Me 307 | 6 | Control | 18±0·6 |
| Me 307 Day 7 | Malignant melanoma | Me 307 | 1 | Control | 101±10 |
| Me 307 | 7 days after | Me 307 | 6 | Control | 16±10 |
| Me 307 | Autoimmunization | Me 307 | 6 | Me 307 Day 0 | 108±13 |
| Me 307 | Autoimmunization | Me 307 | 6 | Me 307 Day 7 | 15±4 |
| Me 307 | Autoimmunization | Me 307 | 6 | Me 307 Day 0 +Day 7 | 104±26 |
| Control | Normal | Me 307 | 1 | Control | 177±11 |
| Control | Normal | Me 307 | 6 | Control | 175±15 |
| Nil | — | Me 307 | — | — | 227±15 |
| Me 312 | Malignant melanoma (recurrent) | Me 312 | 1 | Control | 83±18 |
| Me 312 | Malignant melanoma (recurrent) | Me 312 | 3 | Control | 50±5 |
| Me 312 | Malignant melanoma (recurrent) | Me 312 | 6 | Control | 26±8 |
| Me 312 | Malignant melanoma (recurrent) | Me 312 | 6 | Autologous | 73±11 |

| Lymphocytes | Diagnosis | Target cells | No. of washes | Serum added | Target cells left in well |
|---|---|---|---|---|---|
| Me 313 | Malignant melanoma (metastatic) | Me 312 | 6 | Control | 16±3 |
| Me 313 | Malignant melanoma (metastatic) | Me 312 | 6 | Autologous | 66±12 |
| Me 314 | Malignant melanoma (primary) | Me 312 | 6 | Control | 11±3 |
| Me 314 | Malignant melanoma (primary) | Me 312 | 6 | Autologous | 14±3 |
| Me 315 | Malignant melanoma (primary) | Me 312 | 6 | Control | 28±3 |
| Me 315 | Malignant melanoma (primary) | Me 312 | 6 | Autologous | 19±7 |
| Control | Normal | Me 312 | 1 | Control | 102±7 |
| Control | Normal | Me 312 | 3 | Control | 103±7 |
| Control | Normal | Me 312 | 6 | Control | 87±17 |
| Nil | — | Me 312 | — | — | 103±11 |
| Me 313 | Malignant melanoma (metastatic) | Me 313 | 6 | Control | 149±19 |
| Me 313 | Malignant melanoma (metastatic) | Me 313 | 6 | Autologous | 196±7 |
| Me 314 | Malignant melanoma (primary) | Me 313 | 6 | Control | 76±16 |
| Me 314 | Malignant melanoma (primary) | Me 313 | 6 | Autologous | 62±15 |
| Me 315 | Malignant melanoma (primary) | Me 313 | 6 | Control | 202±7 |
| Me 315 | Malignant melanoma (primary) | Me 313 | 6 | Autologous | 217±15 |
| Control | Normal | Me 313 | 6 | Control | 230±18 |
| Nil | — | Me 313 | — | — | 232±14 |
| Me 313 | Malignant melanoma | Me 314 | 6 | Control | 17±4 |
| Me 313 | Malignant melanoma | Me 314 | 6 | Autologous | 50±6 |
| Me 314 | Malignant melanoma | Me 314 | 6 | Control | 9±4 |
| Me 314 | Malignant melanoma | Me 314 | 6 | Autologous | 13±4 |
| Me 315 | Malignant melanoma | Me 314 | 6 | Control | 63±13 |
| Me 315 | Malignant melanoma | Me 314 | 6 | Autologous | 69±13 |
| Control | Normal | Me 314 | 6 | Control | 98±8 |
| Control | Normal | Me 314 | 6 | Autologous | 99±12 |
| Nil | — | Me 314 | — | — | 122±7 |
| Me 311 | Malignant melanoma (nasal) | Me 311 | 6 | Control | 28±4 |
| Me 311 | Malignant melanoma (nasal) | Me 311 | 6 | Autologous | 55±12 |
| BLA 50 | Bladder carcinoma | Me 311 | 6 | Control | 46±11 |
| BLA 50 | Bladder carcinoma | Me 311 | 6 | Autologous | 41±8 |
| Eth 1 | Ethmoid carcinoma (transitional cell) | Me 311 | 6 | Control | 56±5 |
| Eth 1 | Ethmoid carcinoma (transitional cell) | Me 311 | 6 | Autologous | 60±6 |
| Control | Normal | Me 311 | 6 | Control | 69±8 |
| Control | Normal | Me 311 | 6 | Me 311 | 67±7 |
| Nil | — | Me 311 | — | — | 64±7 |
| Me 318 | Malignant melanoma (disseminated) | Me 318 | 6 | Control | 43±4 |
| Me 318 | Malignant melanoma (disseminated) | Me 318 | 6 | 10% autologous | 124±13 |
| Me 318 | Malignant melanoma (disseminated) | Me 318 | 6 | 5% autologous | 172±18 |
| Me 318 | Malignant melanoma (disseminated) | Me 318 | 6 | 2·5% autologous | 128±11 |
| Me 318 | Malignant melanoma (disseminated) | Me 318 | 6 | 1·25% autologous | 42±7 |
| Me 318 | Malignant melanoma (disseminated) | Me 318 | 6 | HYP 21 | 46±4 |
| HYP 21 | Hypernephroma | Me 318 | 6 | Control | 169±11 |
| HYP 21 | Hypernephroma | Me 318 | 6 | HYP 21 | 167±16 |

FIG. 1.—Effect of increasing number of washes on cytotoxicity of lymphocytes from a patient with extensive malignant melanoma tested on his autologous tumour cells (Me 312). The cytotoxic effect increases with washing and this increase is abolished by the addition of autologous serum.

examine the cross-reactivity of lymphocyte cytolysis on tumours of similar and dissimilar histological diagnosis. Fig. 3 shows the effect of lymphocytes from 4 cases of bladder carcinoma (and one normal) tested on an allogeneic bladder tumour culture after one and 6 washes. It can be seen that there is a cross reaction of lymphocyte kill on bladder tumour cells. The difference in cytotoxicity between minimally washed, and 6 washed, lymphocytes from each case seems to reflect the serum inhibitory activity. A similar cross-reactivity can be seen in cases of malignant melanoma (Table II). These cases also provided an opportunity to compare the cytotoxic effect of the patients' lymphocytes on both autologous and allogeneic melanoma cells. The results indicate that cross reactions in cytotoxicity do occur but also show that such cross reactivity is not universal.

FIG. 2.—Effect of decreasing concentrations of autologous serum on cytotoxicity of well washed lymphocytes on autologous melanoma cells (Case No. Me 318). The inhibitory effect dilutes out at between 1·25 and 2·5% serum.

For instance, the lymphocytes from patient Me 315 were highly cytotoxic on Me 312 cells, but without cytotoxic effect on Me 313 cells, whilst causing only feeble cytolysis on Me 314. Furthermore, autologous cytotoxicity in case Me 313 was less pronounced than that obtained on allogeneic melanoma cells. There was no evidence of cytotoxicity of these patients' lymphocytes on human embryo lung fibroblasts. The cross reacting cytotoxicity of the patients' lymphocytes was however restricted to tumours of similar tissue of origin, *i.e.*, lymphocytes from a patient with bladder carcinoma would not kill either melanoma or fibrosarcoma cells. Furthermore, the specificity of lymphocytes was not decreased by multiple washing, *i.e.*, nonspecific cytotoxicity was not induced.

3. *Inhibitory effect of sera*

The increase in specific cytotoxicity of the patients' lymphocytes with multiple washing would imply that some form of inhibitory factor was being removed. Furthermore, the number of washes rerequired to reveal the cytotoxic effects would perhaps suggest that the inhibitory factor is bound to the lymphocyte surface. The fact that this inhibitor is a serum component was readily demonstrated by adding autologous serum to the washed lymphocytes. Table I shows that the newly acquired cytotoxicity induced by washing of the patients' lymphocytes was abolished by the addition of autologous serum to the reaction mixture. In the few cases where washing increased the nonspecific cytotoxicity of normal allogeneic lymphocytes, the inhibitory

TABLE II.—*Cross-reactivity of the Cytotoxic Effects of Lymphocytes from Patients with Malignant Melanoma on Autologous and Allogeneic Melanoma Cells. The Figures represent Mean Cytotoxicity as a Percentage. CS = with Control Serum. AS = with Autologous Serum*

| | Lymphocytes | | | | | | | | | |
|---|---|---|---|---|---|---|---|---|---|---|
| | Control | | Me 312 | | Me 313 | | Me 314 | | Me 315 | |
| Target cells | CS | AS | CS | AS | CS | AS | CS | AS | CS | AS |
| Me 312 . . . | 1 | 3 | 75 | 29 | 83 | 36 | 89 | 86 | 74 | 82 |
| Me 313 . . . | 1 | 0 | NT | NT | 36 | 16 | 67 | 73 | 13 | 7 |
| Me 314 . . . | 20 | 19 | NT | NT | 86 | 59 | 93 | 89 | 48 | 43 |

FIG. 3.—Cytotoxicity of lymphocytes from 4 cases of bladder carcinoma and one normal control on allogeneic bladder carcinoma cells before and after extensive washing.

sera had no such effect. Furthermore, normal human serum had no effect on the patients' lymphocytes after washing. This serum inhibition appeared to possess specificity for the histological tumour type, *i.e.*, serum from a patient with malignant melanoma would only inhibit lymphocytes from other melanoma patients. Furthermore, an active inhibitor serum from a patient with bladder carcinoma had no effect on the lymphocytes from patients with malignant melanoma.

There were a few cases in which this serum inhibitory activity was undetectable. It is of interest to note that these were cases with a minimal tumour burden, *e.g.*, Me 314 and Me 315, small primary malignant melanomata; those cases with an active serum inhibitory effect were all cases of advanced disease.

4. *Effect of auto-immunization with irradiated cells*

In one case of malignant melanoma (Me 307) with multiple subcutaneous deposits over the arms and trunk, it was decided to test his lymphocytes and serum before and after auto-immunization with his own tumour cells. Several of the subcutaneous lesions were removed surgically and a mechanical cell suspension obtained from them. $5 \times 10^8$ cells were irradiated in a $^{60}Co$ source to a total dose of 12·5 krad and then injected subcutaneously in multiple sites. The studies of his lymphocyte cytotoxicity are shown in Table I. The lymphocytes were tested on cultures of autologous tumour cells.

On Day 0 his lymphocytes were not cytotoxic until washed 6 times, and his serum was inhibitory. On the seventh day after immunization his lymphocytes were powerfully cytotoxic after only one wash, and the Day 7 serum was incapable of preventing this cytotoxicity. It was, however, inhibited by the serum saved from Day 0. In other words the auto-immunization procedure had abolished the serum inhibitory activity although the lymphocytes were still amenable to inhibition by an appropriate serum.

### 5. *Affinity of the serum inhibitory factor*

In the case auto-immunized with his own cells the effect of Day 0 and Day 7 sera on lymphocyte cytotoxicity was examined in 2 ways. Firstly, the sera were incorporated at 5% into the lymphocyte suspensions as described above. They were also tested by pre-incubation of the target cells in 50% serum for one hour at 37°. After washing the wells with culture medium the lymphocytes were then added and their cytotoxicity measured. The results are shown in Table I. The Day 0 serum had a powerful inhibitory effect on cytotoxicity when incorporated into the lymphocyte suspension but was without effect when pre-incubated with the target cells. The Day 7 serum, however, had no inhibitory effect when added to the lymphocytes (at 5%) but when pre-incubated with the target cells (at 50%) produced significant inhibition of lymphocyte killing.

This would suggest that the serum taken before auto-immunization possessed an inhibitory factor with an apparent affinity for the effector cells but with no affinity for the target cells, *i.e.*, it is unlikely to be anti-tumour antibody but is more likely to be circulating antigen. The serum taken after immunization with tumour cells had only a weak inhibiting effect, detectable at high concentration and with affinity for the target cells, *i.e.*, it would seem that the patient's response to immunization was the development of anti-tumour antibody which was responsible for removing the serum inhibitor, antigen.

### DISCUSSION

Our earlier work on the cytotoxicity of lymphocytes from patients with malignant melanoma showed that only lymphocytes from patients with minimal tumour burden were cytotoxic to autologous tumour cells (Currie *et al.*, 1971). The present investigation indicates that this was a somewhat simplified view of the real situation. The majority, perhaps all, of the patients with tumours such as malignant melanoma, bladder carcinoma and hypernephroma have peripheral blood lymphocytes capable of killing autologous tumour cells in tissue culture. The cytotoxic effects were, however, detectable only after extensive washing of the lymphocytes and could be inhibited by the addition of autologous serum but not by normal allogeneic serum. This demonstration of specific serum inhibition of lymphocyte function is in accord with the findings of Hellström *et al.* (1971). In cases with a minimal tumour burden we were unable to detect the serum inhibitory effect. The fact that in the majority of cases with disseminated disease, lymphocyte cytotoxicity was detectable only after multiple washing would suggest that the inhibitory factor was bound to the lymphocyte surface. Since it could be readily washed off the target cells, it is unlikely to be circulating anti-tumour antibody. The abrogation of lymphocyte cytotoxicity by circulating anti-tumour antibody has always been a difficult phenomenon to accept as an important *in vivo* mechanism. The existence of concomitant immunity in the tumour bearing host has always argued against it. However, the possibility that antigen is responsible for the inhibition of lymphocyte function, both local and systemic, is not open to the same criticisms. The correlation of the inhibitory activity of serum with the extent of the disease, and its apparent affinity for the lymphocyte, would also suggest that circulating tumour antigen is a more likely candidate. The fact that autoimmunization with irradiated tumour cells abolished the serum inhibitory activity in the case of malignant melanoma studied, would perhaps indicate that the serum inhibitor (? circulating tumour cell antigen) was cleared from the circulation by antibody (Ikonopisov *et al.*, 1970), thus exposing the cytotoxic effect of the lymphocytes.

The degree of specificity of the lymphocyte cytotoxicity demonstrated in this work is complex. Lymphocytes from the cancer patients did not kill human embryo lung fibroblasts. They did, however, kill the autologous tumour cells and, in many cases, allogeneic tumour cells of similar histological origin. This latter finding would suggest that tumours derived from the same tissue possess similar cross reacting antigens. The extent of such cross reactions is unclear. In this study, especially of cases of melanoma, there was only partial cross reaction between cases. There may also have been a difference in the degree of antigenic expression by cells from different patients. There was no convincing evidence that autologous cytotoxicity was consistently greater than that obtained with allogeneic target cells. The variations in susceptibility to cytotoxic lymphocytes between individual tumours of similar diagnosis may reflect either a quantitative or qualitative difference in their antigenic expression. There is little tangible evidence from this type of study that the target cells possess tumour-associated neo-antigens. The cross reactivity on tumours derived from the same tissue or organ may well reflect autoimmune reactions to organ- or tissue- specific antigens expressed on the surface of the tumour cells. Autoantibodies to many normal tissue components are known to be present in the sera of cancer patients (Whitehouse and Holborow, 1971) and it is conceivable that cell-mediated reactions to organ and tissue specific antigens may masquerade as tumour-specific reactions. A possible explanation for the cross reactivity is that malignant transformation is associated with exposure of an antigen on the cell surface, which is normally present but shielded by some material and therefore not " expressed " on the normal tissue. The malignant change would, therefore, not consist of the synthesis of a neo-antigen but a change which allows an existing macromolecule to be expressed and detected by the host's immunological apparatus.

The demonstration of serum factors capable of specifically inhibiting the *in vitro* cytotoxic properties of the patient's lymphocytes raises the question of the role, if any, of such factors *in vivo*. If, as we have postulated, they are solubilized tumour antigens in one form or another, then the constant release of such potential inhibitors of cell-mediated immunity from the tumour cell surface could be held to play a vital role in the natural history of immune reactions to tumours. Alexander and his colleagues (1967) have shown that the presence of a small limb tumour in the rat effectively paralyses the regional draining lymph nodes, inhibiting the propagation of specific cell-mediated responses from the nodes. The most convenient explanation of such a phenomenon would seem to be local inhibition of the node lymphoid cells by their persistent exposure to low concentrations of tumour antigen. As the tumour progresses and the local node is overwhelmed, then antigen may well reach the circulation and its concentration there increase with tumour growth. In this way central inhibition of lymphocyte function by tumour antigens could be held to account for both local and systemic abrogation of immune responses.

In the design of any immunological treatment of human tumours, the effect of any potential therapy on serum inhibitory activity will obviously have to be borne in mind. We have described here a single case in which auto-immunization with tumour cells abolished the patient's serum inhibitory activity. In our previous series of patients treated in this way, 5 out of 12 cases " developed " cytotoxic lymphocytes following immunization (Currie *et al.*, 1971). In the light of the present experiments, it would seem that these cases may well represent the disappearance of serum inhibitors. Bansal and Sjögren (1971) have suggested that the ability of an " immune " serum to remove blocking activity from tumour bearing serum may well correlate with its *in vivo* anti-tumour effects. They treated rats bearing polyoma tumours with these so-called " de-blocking sera " and were able to induce complete tumour regression. The mechanism of this regression was unclear. It is feasible that it was merely the cytotoxic effect of the sera on the target cells. However, should this phenomenon prove to be due to the " de-blocking " activity of the serum, then this experiment would indicate that the serum inhibitory activity plays an important, even crucial, role in inhibiting tumour rejection.

Further investigation of the nature and activity of these tumour-associated serum factors and their correlation with clinical status will help to elucidate their role *in vivo*. Should they prove to be important specific *in vivo* moderators of cell-mediated immunity in cancer patients, then they provide a distinct hope for immunotherapeutic techniques. Such serum factors are readily detectable, even quantitatible entities, and the measurement of changes in them would provide a valuable means of monitoring any form of immunological treatment. Quantitation of serum inhibitory factors may also be of value in assessing tumour burden. If they reflect the extent of the disease, then they may be useful both for measuring the effects of treatment and for early detection of recurrence.

This communication also draws attention to a potential source of error in any studies of lymphocyte cytotoxicity of tumour cells *in vitro*. The method of preparation of the lymphocytes and the degree of washing they receive appears to be a crucial variable which must be borne in mind when drawing any conclusions about cytotoxicity and the effects of serum components.

This work has been supported by grants made to the Chester Beatty Research Institute by the Cancer Research Campaign and the Medical Research Council.

We thank Professors P. Alexander and G. Hamilton Fairley for their invaluable advice and encouragement. We also wish to thank our surgical colleagues, especially Mr C. I. Cooling and Mr D. M. Wallace, for their enthusiastic co-operation. The technical assistance of Mr M. Lovell is gratefully acknowledged.

### REFERENCES

Alexander, P., Bensted, J., Delorme, E. J., Hall, J. G. & Hodgett. J. (1967) The Cellular Immune Response to Primary Sarcomata in Rats. II. Abnormal Responses of Nodes Draining the Tumour. *Proc. R. Soc.*, B, **174**, 237.

Bansal, S. C. & Sjögren, H. O. (1971) " Unblocking " Serum Activity *in vitro* in the Polyoma System may Correlate with Anti-tumour Effects of Antiserum *in vivo*. *Nature, Lond.*, **233**, 76.

Currie, G. A., Lejeune, F. & Fairley, G. H. (1971) Immunization with Irradiated Tumour Cells and Specific Lymphocyte Cytotoxicity in Malignant Melanoma. *Br. med. J.*, ii, 305.

Gold, P. & Freedman, S. O. (1965) Specific Carcinoembryonic Antigens of the Human Digestive System. *J. exp. Med.*, **122**, 467.

Hellström, I., Hellström, K. E., Sjögren, H. D. & Warner, G. A. (1971) Demonstration of Cell-mediated Immunity to Human Neoplasms of Various Histological Types. *Int. J. Cancer*, **7**, 1.

Ikonopisov, R. L. *et al.* (1970) Auto-immunization with Irradiated Tumour Cells in Human Malignant Melanoma. *Br. med. J.*, ii, 752.

SJÖGREN, H. O., HELLSTRÖM, I., BANSAL, S. C. & HELLSTRÖM, K. E. (1971) Suggestive Evidence that the "Blocking Antibodies" of Tumour-bearing Individuals may be Antigen–Antibody Complexes. *Proc. U.S. Acad. Sci.*, **68**, 1372.

WHITEHOUSE, J. M. A. & HOLBOROW, E. J. (1971) Smooth Muscle Antibody in Malignant Disease. *Br. med. J.*, i,, 511.